# Short-form video addiction and academic burnout in adolescence: the mediating role of emotional state and the moderating role of grade level

**DOI:** 10.3389/fpsyg.2026.1864012

**Published:** 2026-08-26

**Authors:** Qing Wang, Huiting Li, Feng Li, Huaqiang Liu, Zhensong Lan, Shi Yu

**Affiliations:** 1 School of Sociology and Humanities, Jiangxi University of Finance and Economics, Nanchang, China; 2 Collaborative Innovation Center of Assessment for Basic Education, Beijing Normal University-Zhuhai Campus, Zhuhai, China; 3 Key Laboratory of Digital Analysis and Intelligent Decision-Making for Urban–Rural Industrial Integration Development, Sichuan Province for Philosophy and Social Sciences, School of Law and Public Administration, Yibin University, Yibin, Sichuan, China; 4 School of Humanities and Social Sciences, Guangxi Medical University, Nanning, China

**Keywords:** academic burnout, adolescents, emotional state, family intimacy, grade level, short-form video addiction

## Abstract

**Objective:**

This study examined the relationship between short-form video addiction and academic burnout among adolescents, focusing on the mediating role of emotional state and the moderating role of grade level. The potential moderating role of family intimacy was also explored.

**Methods:**

A sample of 1,719 secondary school students (51.9% male) from five provinces in China completed validated measures of short-form video addiction, emotional state (DASS-21), family intimacy (Family Environment Scale), and academic burnout. Moderated mediation analyses were conducted using linear regression with bootstrapped confidence intervals.

**Results:**

Short-form video addiction positively predicted academic burnout (β = 0.162, *p* < 0.001). Emotional state partially mediated this association [indirect effect = 0.034, 95% bootstrap *CI* (0.022, 0.046)], accounting for 17.3% of the total effect. Grade level significantly moderated the direct path (β = −0.024, *p* = 0.009), such that the association between addiction and burnout decreased with advancing grade level. The interaction between emotional state and family intimacy was not statistically significant (β = 0.012, *p* = 0.334). The interaction between addiction and family intimacy was significant (β = −0.026, *p* = 0.039).

**Conclusion:**

Grade level significantly attenuated the direct risk of short-form video addiction, whereas family intimacy did not reliably moderate the emotional pathway. Interventions targeting emotion regulation skills and considering developmental stage may be promising for reducing academic burnout among adolescents.

## Introduction

Adolescence is a critical developmental period during which academic engagement serves as a key indicator of psychosocial adjustment and long-term educational attainment. Academic burnout is a maladaptive syndrome characterized by emotional exhaustion, cynical detachment from schoolwork, and a diminished sense of scholastic competence (Wu et al., 2007). Longitudinal evidence has shown that elevated burnout predicts subsequent truancy, absenteeism, and a range of internalizing symptoms including anxiety and depression (Chae, 2015; Wang et al., 2015). Therefore, understanding the mechanisms that initiate and sustain academic burnout is essential for developing evidence-based prevention and intervention strategies tailored to adolescents.

The proliferation of algorithm-driven short-form video platforms, such as TikTok and Douyin, has positioned these applications as a dominant media format among adolescents (Kaye et al., 2021). Although prior research has linked general smartphone overuse to poorer academic outcomes (Xu et al., 2023; He and An, 2022), the specific pathways through which short-form video addiction contributes to academic burnout remain underexplored. This form of behavioral addiction is characterized by compulsive usage, loss of control, tolerance (i.e., increasing usage time to achieve the same level of satisfaction), and withdrawal symptoms when use is restricted (Li et al., 2021). The intermittent, variable-ratio reinforcement schedules embedded in short-video platforms are theorized to foster addictive patterns by exploiting basic learning mechanisms (Skinner, 1953). However, such accounts often neglect the affective mechanisms that may explain how addiction translates into academic dysfunction, as well as the contextual factors, particularly family environment, that may buffer this process.

Theorists have long recognized the centrality of affective processes in both addictive behaviors and academic functioning. According to the Interaction of Person-Affect-Cognition-Execution (I-PACE) model (Brand et al., 2016), individuals may develop addictive behaviors as maladaptive coping strategies in response to negative emotional states. In the context of short-form video addiction, adolescents experiencing negative affect, such as depression, anxiety, or stress, may be particularly vulnerable to compulsive viewing, which in turn reinforces these emotional disturbances. Concurrently, research in educational psychology has established robust associations between negative emotional states and academic burnout (Wu et al., 2024; Slivar, 2001). Specifically, depression diminishes interest and energy for academic tasks; anxiety impairs concentration and fosters avoidance; and stress depletes self-regulatory resources (Lazarus and Folkman, 1984). Integrating these lines of inquiry, it is proposed that emotional state serves as a key mediator linking short-form video addiction to academic burnout: addiction may intensify negative affect, which subsequently erodes academic engagement and accelerates burnout.

The family context represents a critical microsystem that shapes adolescents' emotional experiences and behavioral responses (Bronfenbrenner, 1979). Among family characteristics, family intimacy is defined as the degree of emotional bonding, warmth, and responsive communication among members. It has been identified as a potentially protective factor (Sheng et al., 2023). High family intimacy may buffer the adverse consequences of short-form video addiction by providing an immediate context for emotional regulation. Specifically, even when adolescents experience negative affect following compulsive viewing, cohesive families can offer shared activities, open communication, and emotional support. These resources help neutralize distress and prevent its translation into academic disengagement (Tian, 2023). Conversely, in low-intimacy families, adolescents may lack such resources, leaving them more vulnerable to the cascade from negative affect to burnout. This reasoning suggests that family intimacy may moderate the latter stage of the indirect pathway, specifically the link from emotional state to academic burnout, as well as the direct link between addiction and burnout.

Developmentally, the impact of digital media on academic outcomes may vary across adolescence due to changes in self-regulation, peer influence, and academic demands. Older adolescents typically possess better cognitive control and may have developed more effective coping strategies (Steinberg, 2008). Therefore, grade level, as a proxy for developmental stage, may moderate the direct association between short-form video addiction and academic burnout, with the relationship weakening in higher grades. Although prior studies have rarely examined grade as a moderator in this specific mediation model, exploratory evidence suggests that media effects on school performance can differ between middle and high school students (Xu et al., 2023). Including grade level as an additional moderator can provide a more nuanced understanding of how the risk of short-form video addiction changes across adolescence. In the present study, grade level was treated as a continuous variable for moderation analyses, as the six-grade levels allowed for linear trend estimation.

### The present study and hypotheses

Based on the theoretical framework outlined above, the present study tested a moderated mediation model to examine the relationship between short-form video addiction and academic burnout among adolescents, with emotional state as a mediator and family intimacy and grade level as moderators. Specifically, the following hypotheses were proposed:

Hypothesis 1 (H1): Short-form video addiction is positively associated with academic burnout.
Hypothesis 2 (H2): Emotional state mediates the relationship between short-form video addiction and academic burnout.
Hypothesis 3a (H3a): Family intimacy moderates the direct relationship between short-form video addiction and academic burnout.
Hypothesis 3b (H3b): Family intimacy moderates the second stage of the indirect pathway (emotional state → academic burnout).
Hypothesis 4 (H4): Grade level moderates the direct relationship between short-form video addiction and academic burnout.

By identifying the conditions under which short-form video addiction most strongly predicts academic burnout, this study aims to provide parents and educators with actionable insights for developmentally sensitive interventions in the digital age.

## Materials and methods

### Participants

This study employed cluster sampling to select participants from seven middle and high schools across five provinces in China: Henan, Shanxi, Guizhou, Shandong, and Jiangxi. A total of 1,765 students were initially recruited. Questionnaires exhibiting a highly consistent response pattern, obvious logical errors, or more than four consecutive missing items were excluded. The final valid sample comprised 1,719 participants, yielding an effective response rate of 97.4%. Grade distribution was as follows: 310 first-year junior high school students (18.0%), 279 second-year junior high students (16.2%), 352 third-year junior high students (20.5%), 285 first-year senior high students (16.6%), 262 second-year senior high students (15.2%), and 231 third-year senior high students (13.4%). Of the participants, 892 were male (51.9%) and 827 were female (48.1%). The sample was predominantly Han Chinese (over 95%), reflecting the regional demographics.

### Measures

#### Short-form video addiction

The Short-Form Video Addiction Scale (You et al., 2022) was used to assess addictive tendencies toward short-form video platforms. The scale consists of seven items rated on a five-point Likert scale ranging from zero (strongly disagree) to four (strongly agree). Sample items include “I feel addicted to short videos.” All items were positively scored, with higher total scores indicating greater addiction severity. In this study, the scale demonstrated good reliability, with a Cronbach's α of 0.81. A confirmatory factor analysis (CFA) showed suboptimal fit (CFI = 0.84, RMSEA = 0.136, SRMR = 0.072). Despite this, the total score had acceptable reliability (α = 0.81) and was retained for analysis, given its common use in applied research.

#### Emotional state

The Depression, Anxiety, and Stress Scale-21 (DASS-21; Henry and Crawford, 2005) was used to measure negative emotional states. The scale comprises 21 items divided into three subscales: depression, anxiety, and stress, each containing seven items. Responses are rated on a four-point Likert scale ranging from zero (does not apply) to three (always applies). Higher total scores indicate greater emotional distress. In this study, the scale showed excellent internal consistency (Cronbach's α = 0.94). A three-factor CFA showed acceptable fit (CFI = 0.90, RMSEA = 0.075, SRMR = 0.046), but the latent correlation between anxiety and stress was very high (*r* = 0.98), indicating poor discriminant validity. Therefore, we used the total score as a composite measure. Given the high correlation between anxiety and stress, the total score was used as a general indicator of negative emotional state rather than as three separate constructs.

#### Academic burnout

The Academic Burnout Scale was adapted from the Learning Situation Scale (Dai, 2015). A total of 16 items were used, covering three dimensions: emotional exhaustion, academic disengagement, and reduced sense of achievement. Some items (e.g., “I can study with energy”) were reverse-scored. All items were rated on a five-point scale ranging from zero (completely disagree) to four (completely agree). Higher total scores indicate higher levels of academic burnout. The scale has shown adequate psychometric properties in previous Chinese adolescent studies (Wu et al., 2007). In this study, the scale had a Cronbachs α of 0.82. A three-factor CFA yielded suboptimal fit (CFI = 0.81, RMSEA = 0.096, SRMR = 0.116), reflecting the multidimensional nature of academic burnout. Despite this suboptimal fit, the total score demonstrated acceptable reliability (α = 0.82) and was retained for subsequent analyses.

#### Family intimacy

The Family Environment Scale (Moos, 1974; Chinese version by Fei et al., 1991) was used, and only the Cohesion subscale was administered. Six items (e.g., “Family members always give each other the greatest help and support”) were rated on a five-point scale from zero (completely disagree) to four (completely agree). Higher scores indicate greater family intimacy. In this sample, the scale had a Cronbach's α of 0.80. A one-factor CFA showed good fit (CFI = 0.957, RMSEA = 0.078, SRMR = 0.037).

#### Demographic variables

Participants reported their grade level, gender, and left-behind status (whether one or both parents had worked away from home for more than 6 months in the past year).

#### Data analysis

Data were analyzed using SPSS 24.0 (IBM Corp., Armonk, NY, USA) and R 4.4 (R Core Team, Vienna, Austria). First, Harman's single-factor test was used to examine common method bias. Second, descriptive statistics and Pearson correlations were computed for all core variables. Third, a moderated mediation model equivalent to PROCESS Model 59 (Hayes, 2018) was tested using linear regression. Short-form video addiction was the independent variable, academic burnout the dependent variable, emotional state the mediator, and family intimacy and grade level (centered) were specified as moderators. Grade level was treated as a continuous variable, as the six-grade levels allowed for linear trend estimation. The model estimated the moderation of the first stage (addiction → emotion), the second stage (emotion → burnout), and the direct effect (addiction → burnout). All continuous variables were standardized prior to analysis. Indirect effects and their 95% confidence intervals were estimated using non-parametric bootstrapping with 5,000 resamples. Gender, grade level (when not serving as a moderator), and left-behind status were included as covariates in all models.

## Results

### Common method bias test

Harman's single-factor test was conducted to examine common method bias (Harman, 1967). The results indicated that 19 factors with eigenvalues greater than one were extracted without rotation, with the first factor accounting for 14.97% of the variance, which is below the recommended threshold of 40%. Thus, common method bias did not significantly affect the data.

### Descriptive statistics and correlations

Descriptive statistics and bivariate correlations are presented in Table 1. Short-form video addiction was positively correlated with emotional state (*r* = 0.19, *p* < 0.001) and academic burnout (*r* = 0.31, *p* < 0.001), and negatively correlated with family intimacy (*r* = −0.17, *p* < 0.001). Emotional state was positively correlated with academic burnout (*r* = 0.43, *p* < 0.001) and negatively correlated with family intimacy (*r* = −0.25, *p* < 0.001). Family intimacy was negatively correlated with academic burnout (*r* = −0.73, *p* < 0.001). Grade level was negatively correlated with academic burnout (*r* = −0.14, *p* < 0.001) and positively correlated with family intimacy (*r* = 0.16, *p* < 0.001). These preliminary correlations support Hypothesis 1.

**Table 1:** Descriptive statistics and correlations among key variables (*N* = 1,719).

Variable	*M*	*SD*	1	2	3	4	5
1. Short-form video addiction	16.67	4.97	—				
2. Academic burnout	34.23	8.67	0.31^***^	—			
3. Emotional state	46.26	12.38	0.19^***^	0.43^***^	—		
4. Family intimacy	12.33	4.20	−0.17^***^	−0.73^***^	−0.25^***^	—	
5. Grade level	2.35	1.66	0.02	−0.14^***^	0.08^**^	0.16^***^	—

Grade level was coded as 0 = first-year junior high, 1 = second-year junior, 2 = third-year junior, 3 = first-year senior, 4 = second-year senior, 5 = third-year senior. ^**^p < 0.01. ^***^p < 0.001.

### Moderated mediation model testing

All continuous variables were standardized prior to analysis. The moderated mediation model was tested using linear regression with bootstrapped confidence intervals (5,000 resamples). Results are presented in Table 2.

**Table 2:** Moderated mediation model results (standardized coefficients).

Predictor	Emotional state (ES)	Academic burnout (AB)
SFVA	0.139^***^	0.162^***^
FI	−0.236^***^	−0.629^***^
ES	—	0.243^***^
SFVA × FI	0.009	−0.026^*^
ES × FI	—	0.012
Grade	0.062^***^	−0.040^***^
SFVA × Grade	−0.002	−0.024^**^
ES × Grade	—	0.004
Gender	−0.016	0.027
Left-behind	0.164^**^	0.016
*R* ^2^	0.100	0.625

All continuous variables were standardized. Grade level was centered. SFVA, short-form video addiction; FI, family intimacy; ES, emotional state; AB, academic burnout. Bootstrap confidence intervals (percentile method) based on 5,000 resamples. ^*^p < 0.05. ^**^p < 0.01. ^***^p < 0.001.

#### Emotional state model

Short-form video addiction positively predicted emotional state (β = 0.139, *p* < 0.001), and family intimacy negatively predicted emotional state (β = −0.236, *p* < 0.001). The interaction term between addiction and family intimacy was not significant (β = 0.009, *p* = 0.624), nor was the interaction between addiction and grade level (β = −0.002, *p* = 0.908). Grade level itself positively predicted emotional state (β = 0.062, *p* < 0.001). The model explained 10.0% of the variance in emotional state.

##### Academic burnout model

**Figure 1:**
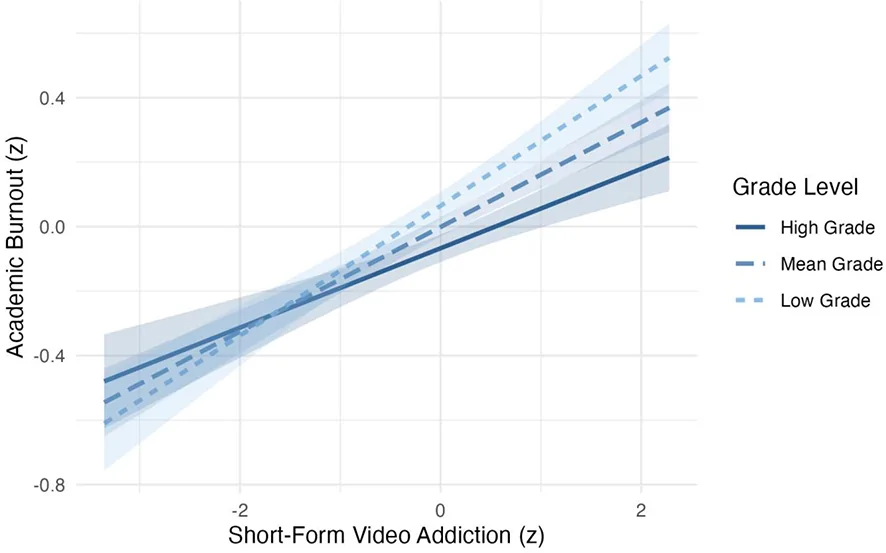
Grade level moderates the direct relationship between short-form video addiction and academic burnout. Simple slopes are plotted at low (−1 *SD*), mean, and high (+1 *SD*) levels of grade level. The positive association between addiction and burnout was weaker at higher grade levels, but the effect size was very small (β = −0.024, *p* = 0.009).

Short-form video addiction positively predicted academic burnout [β = 0.162, *p* < 0.001; 95% bootstrap *CI* (0.132, 0.192)], supporting Hypothesis 1. Emotional state also positively predicted burnout (β = 0.243, *p* < 0.001), while family intimacy negatively predicted burnout (β= −0.629, *p* < 0.001). The interaction between emotional state and family intimacy was not significant [β = 0.012, *p* = 0.334; 95% bootstrap *CI* (−0.013, 0.037)], indicating that family intimacy did not moderate the second stage of the indirect pathway. The interaction between addiction and grade level was significant [β = −0.024, *p* = 0.009; 95% bootstrap *CI* (−0.041, −0.006)], indicating that grade level moderated the direct path. Simple slope analysis revealed that the association between addiction and burnout was weaker at higher grade levels (+1 *SD*: slope = 0.15) than at lower grade levels (−1 *SD*: slope = 0.20). The effect size was very small (β = −0.024), suggesting limited practical significance (Figure 1). The interaction between addiction and family intimacy was also significant [β = −0.026, *p* = 0.039; 95% bootstrap *CI* (−0.051, −0.001)]. The interaction between emotional state and grade level was not significant (β = 0.004, *p* = 0.666). The model explained 62.5% of the variance in academic burnout.

The indirect effect of short-form video addiction on academic burnout through emotional state was significant [indirect effect = 0.034, 95% bootstrap *CI* (0.022, 0.046)], supporting Hypothesis 2. The total effect of addiction on burnout was 0.196 (0.162 direct + 0.034 indirect). Thus, the indirect effect accounted for 17.3% of the total effect. When examining different levels of family intimacy (mean ± 1 *SD*), the indirect effect was 0.037 for low intimacy and 0.030 for high intimacy.

## Discussion

This study examined the mediating role of emotional state and the moderating roles of family intimacy and grade level in the relationship between short-form video addiction and academic burnout among Chinese adolescents. The findings yielded several important insights, organized around the four sets of hypotheses.

### Direct effect of short-form video addiction on academic burnout

Consistent with Hypothesis 1, short-form video addiction was positively associated with academic burnout. This result aligns with previous research on problematic media use and academic outcomes (Xu et al., 2023; Qu et al., 2017). The association remained robust after controlling for gender, grade, and left-behind status. The algorithmic recommendation mechanisms of short-form video platforms deliver highly tailored content that triggers intense excitement and consumes substantial cognitive resources (Christakis et al., 2004), leading to attentional shifts and physical-mental exhaustion. Additionally, addicted adolescents may spend excessive time online, reducing engagement in academic tasks (Singh and Srivastava, 2021) and fostering academic procrastination (Xie et al., 2023). Collectively, these processes may contribute to a diminished sense of academic achievement and accelerated burnout.

### Mediating role of emotional state

Supporting Hypothesis 2, emotional state significantly mediated the relationship between short-form video addiction and academic burnout. Specifically, addiction was associated with more negative emotional states, which in turn were associated with higher burnout. This finding is consistent with the I-PACE model (Brand et al., 2016). The Conservation of Resources Theory (Hobfoll, 1989) provides a complementary explanation: negative emotions consume psychological resources such as attention, self-control, and motivation, thereby impairing cognitive functioning and academic performance. Although the cross-sectional design precludes causal inferences, the mediation pattern is theoretically plausible and aligns with longitudinal evidence on media use and emotional maladjustment.

### Moderating role of family intimacy

Hypothesis 3b predicted that family intimacy would moderate the second stage of the indirect pathway (emotional state → burnout). The interaction between emotional state and family intimacy was not statistically significant [β = 0.012, *p* = 0.334; 95% bootstrap *CI* (−0.013, 0.037)]. Thus, Hypothesis 3b was not supported.

The interaction between addiction and family intimacy was statistically significant [β = −0.026, *p* = 0.039; 95% bootstrap *CI* (−0.051, −0.001)], indicating that family intimacy moderated the direct path. However, the direction of this effect was not consistent with the hypothesized buffering pattern, warranting further investigation.

### Moderating role of grade level

Hypothesis 4 was supported: grade level significantly moderated the direct relationship between short-form video addiction and academic burnout, with the association being weaker at higher grade levels. This developmental finding suggests that as adolescents grow older, the academic risk of short-form video addiction diminishes. Possible explanations include improved self-regulation, better time management skills, or increased academic pressure that may override the relative impact of media use. Although the interaction was statistically significant, the effect size was very small (β = −0.024), indicating that the practical significance of this moderation is limited. This result underscores the importance of considering developmental stage when designing interventions; younger adolescents may benefit more from family-based support, whereas older adolescents might respond to self-regulation training.

### Theoretical and practical contributions

Theoretically, this study integrates family intimacy and grade level into the short-form video addiction-emotion-burnout chain, refining the understanding of how contextual and developmental factors shape the translation of addictive behaviors into academic outcomes. The finding that family intimacy did not reliably moderate the emotional-to-burnout pathway suggests that family factors may operate through other mechanisms. Practically, the mediating role of emotional state suggests that interventions targeting academic burnout should incorporate emotion regulation skills training, especially for students exhibiting signs of addiction. Furthermore, the grade-level moderation indicates that prevention efforts should be tailored to developmental stage: earlier interventions may focus on family-based monitoring and support, while later interventions may emphasize self-regulation and coping strategies.

## Limitations and future directions

Several limitations should be acknowledged. First, the cross-sectional design precludes causal inferences. Although the theoretical model posits short-form video addiction as a predictor of academic burnout, bidirectional relationships are plausible. Future research should employ longitudinal designs to establish temporal precedence. Second, data were collected via self-report questionnaires, which may be subject to social desirability and common method bias. Although Harman's single-factor test suggested this bias was not a major concern, future studies should incorporate objective measures (e.g., screen time tracking) or multi-informant reports. Third, cluster sampling was employed across seven schools, but school identifiers were not recorded. Consequently, the study was unable to adjust for potential within-school clustering. Future research should retain school identification codes to enable more rigorous statistical control. Fourth, the emotional state model explained only 10% of the variance, indicating that short-form video addiction accounts for a relatively small portion of negative affect. Unmeasured variables likely play larger roles. Fifth, confirmatory factor analyses showed suboptimal fit for the short-form video addiction scale and the academic burnout scale.

## Conclusion

Based on a sample of 1,719 Chinese secondary school students, the present study demonstrated that short-form video addiction is positively associated with academic burnout, and that emotional state partially mediates this relationship. Grade level significantly moderated the direct association between addiction and burnout, with the relationship weakening at higher grades, although the effect size was very small. The moderating role of family intimacy on the emotional-to-burnout pathway was not supported. These findings suggest that interventions aiming to reduce academic burnout should target emotional regulation skills and consider developmental stage, whereas the role of family intimacy remains uncertain based on the current data. Future longitudinal research is needed to establish causality and to examine how these mechanisms unfold over time.

## Data Availability

The original contributions presented in the study are included in the article/supplementary material, further inquiries can be directed to the corresponding authors.
